# Glucocorticoid Receptor, but Not Mineralocorticoid Receptor, Mediates Cortisol Regulation of Epidermal Ionocyte Development and Ion Transport in Zebrafish (*Danio Rerio*)

**DOI:** 10.1371/journal.pone.0077997

**Published:** 2013-10-29

**Authors:** Shelly Abad Cruz, Chia-Hao Lin, Pei-Lin Chao, Pung-Pung Hwang

**Affiliations:** Institute of Cellular and Organismic Biology, Academia Sinica, Nankang, Taipei, Taiwan, R. O. C; Baylor College of Medicine, United States of America

## Abstract

Cortisol is the major endogenous glucocorticoid (GC) both in human and fish, mediated by corticosteroid receptors. Due to the absence of aldosterone production in teleost fish, cortisol is also traditionally accepted to function as mineralocorticoid (MC); but whether it acts through the glucocorticoid receptor (GR) or the mineralocorticoid receptor (MR) remains a subject of debate. Here, we used loss-of-function and rescue assays to determine whether cortisol affects zebrafish epidermal ionocyte development and function via the GR and/or the MR. GR knockdown morphants displayed a significant decrease in the major ionocytes, namely Na^+^-K^+^-ATPase-rich cells (NaRCs) and H^+^-ATPase-rich cells (HRCs), as well as other cells, including epidermal stem cells (ESCs), keratinocytes, and mucus cells; conversely, cell numbers were unaffected in MR knockdown morphants. In agreement, GR morphants, but not MR morphants, exhibited decreased NaRC-mediated Ca^2+^ uptake and HRC-mediated H^+^ secretion. Rescue via GR capped mRNA injection or exogenous cortisol incubation normalized the number of epidermal ionocytes in GR morphants. We also provide evidence for GR localization in epidermal cells. At the transcript level, GR mRNA is ubiquitously expressed in gill sections and present in both NaRCs and HRCs, supporting the knockdown and functional assay results in embryo. Altogether, we have provided solid molecular evidence that GR is indeed present on ionocytes, where it mediates the effects of cortisol on ionocyte development and function. Hence, cortisol-GR axis performs the roles of both GC and MC in zebrafish skin and gills.

## Introduction

Glucocorticoid (GC) was discovered more than a century ago, and the GC cortisol plays a key role in carbohydrate metabolism in mammals [1]. Cortisol action is mediated by two corticosteroid receptors (CRs): the glucocorticoid receptor (GR) and the mineralocorticoid (MC) receptor (MR) [2], [3]. The MR also binds aldosterone with similar affinity, but cortisol is less potent at inducing transactivation of MR; despite of this, cortisol is the major ligand of MR in several organs [4], [5]. The ability of cortisol to target both CRs complicates elucidation of the pathways by which cortisol affects biological processes. The two CRs are steroid receptors of the nuclear receptor (NR) superfamily, and have several similar physiological functions [6]. Despite these similarities, their respective ligands retain distinct roles [2]: GC cortisol affects cardiovascular function, immune/stress response, cell cycle, growth, reproduction, and brain-related neuronal activities [7]–[9], whereas MC aldosterone affects osmoregulation and acid-base homeostasis [10], [11]. Previous extensive studies provide a comprehensive platform on mammalian corticosteroid system. Other vertebrates benefits from this knowledge, but the absence of MC production in fish entails a different scheme of cortisol signaling pathway.

Cortisol in teleost fish shares several biological roles with its mammalian equivalent [12], [13], but whether it exerts these effects as being GC and/or MC is an important question from both a comparative and evolutionary physiological point of view. Unlike mammals, teleost fish do not possess aldosterone [14], [15], and several studies have provided evidence that teleost cortisol performs a compensatory role through MR [16]–[18]. On the other hand, fish have been reported to contain 11-deoxycorticosterone (DOC), a precursor molecule for the production of aldosterone [15]. DOC, being a potent agonist of MR, was implied to be the MC equivalent in fish [18], [19]. However, the low DOC plasma level in rainbow trout has led to the suggestion that cortisol is still the primary requirement for the osmoregulatory role of MR in teleosts [20].

Several studies have investigated the role of cortisol with GR and/or MR in fish osmoregulation, primarily through pharmacological approaches; however, some of the results are conflicting [20]–[26]. Those inconsistent results may have arisen from technical limitations or differences in the experimental designs and/or the model systems used, and pharmacological approaches alone are apparently insufficient at resolving the exact pathway by which cortisol exerts its action on fish osmoregulation. A more suitable model may be zebrafish, which has been used for the past thirty years to address scientific questions with straightforward molecular technologies [27], [28]. Hence, the use of zebrafish may allow us to delineate the roles of GR and MR during epidermal ionocyte development, in relation to their effects on osmo/ionoregulation. Collectively, the previous studies suggest that cortisol coordinates a primary adaptive response in seawater and freshwater fishes, while specialized epithelial cells (ionocytes, a.k.a. chloride cells) in skin/gills play a major role in regulating osmo- and iono-regulation [21], [22], [29], [30]. In fact, cortisol has been linked to cell differentiation and proliferation, based on the observed increase and morphological changes in ionocytes during environmental acclimation of teleost fish [31]–[39]. Using the established ionocyte development model platform [40]–[43], we recently proved that cortisol regulates zebrafish ionocyte differentiation through the Foxi3a/−b transcription factors [44]. In addition, gene knockdown of *gr*, but not *mr*, has been shown to diminish the function of epithelial calcium channels (ECaCs), which are known to be expressed in Na^+^-ATPase-rich cell (NaRC) ionocytes [45]. A separate group reported that knockdown of zebrafish *gr* caused decreased Na^+^-uptake via H^+^-ATPase-rich cells (HRCs), and substantiated their results with treatment of GR agonist or antagonist; aldosterone treatment, on the other hand, did not affect Na^+^-uptake [26]. Taken together, it appears that cortisol may control epidermal ionocyte development and function through GR alone. Testing this hypothesis may enable the specific contributions of GR and MR to be further defined.

Following our recent report that exogenous cortisol promotes epidermal ionocyte progenitor differentiation in zebrafish [44], here we attempt to extend our understanding of the mechanism of cortisol action. We therefore designed experiments to determine whether GR and/or MR mediate the effects of cortisol on ionocyte development and function. To this end, we performed gene knockdown and rescue experiments with morpholino oligonucleotides against the *gr* and *mr* genes, and examined *GR* mRNA expression in epidermal cells (especially ionocytes) to confirm its role in development and iono-regulation.

## Results

### The Effect of GR and MR Knockdown on the Density of Ionocytes

**Figure 1:**
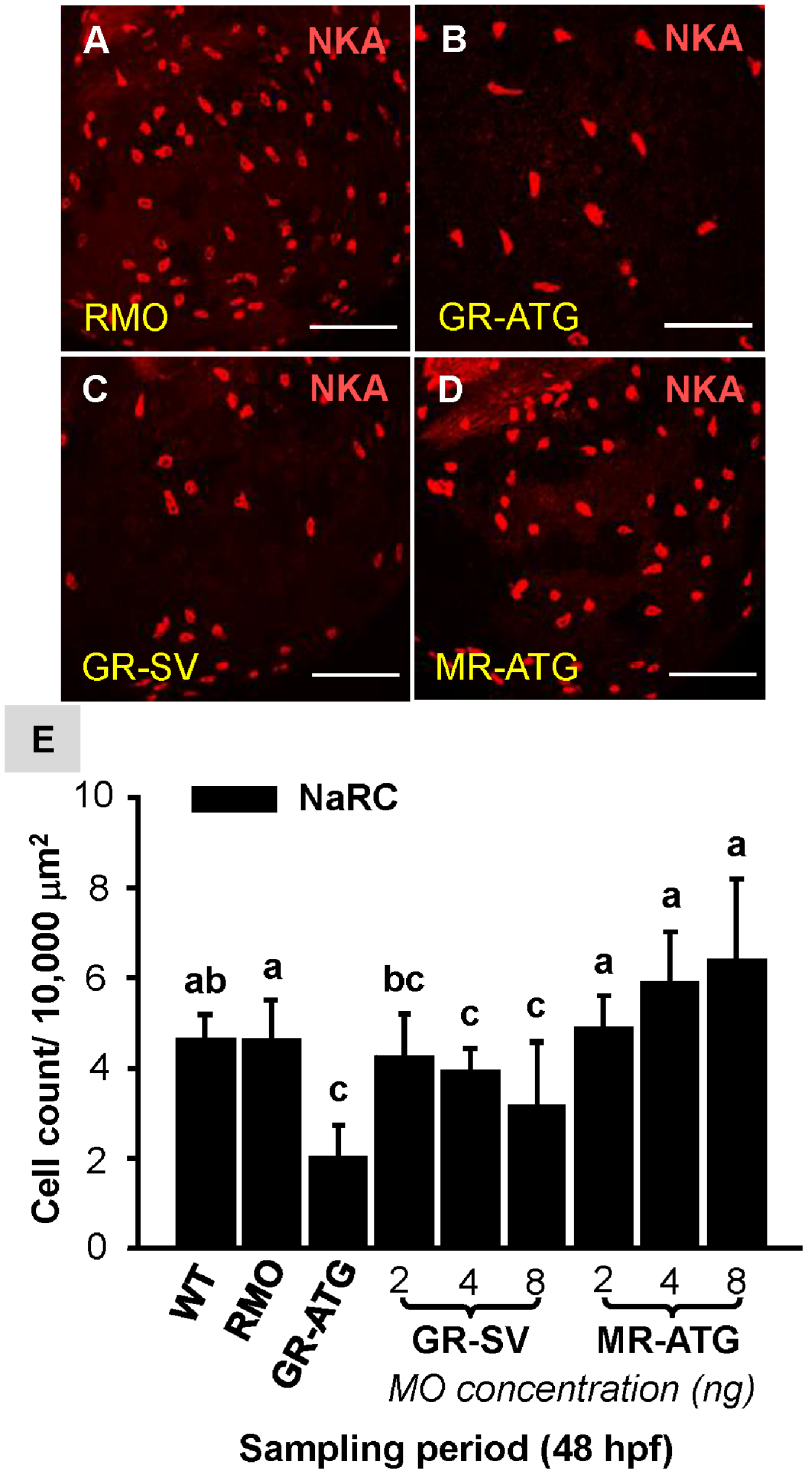
Effect of corticosteroid receptor gene knockdown on NaRC number. Zebrafish embryos at the 1∼4 cell-stage were microinjected with glucocorticoid receptor ATG-MO (GR-ATG), GR-splice variant MO (GR-SV), mineralocorticoid receptor ATG-MO (MR-ATG), or Random-MO (RMO; control). Representative images of yolk-sac NaRCs labeled with anti-α sub-unit of Na^+^-K^+^-ATPase (NKA) in RMO (*A*), GR-ATG (*B*), GR-SV (*C*), and MR-ATG (*D*) morphants. NaRC numbers are compared in (*E*). Values are presented as the mean ±s.d. (n = 10–12). ^ab^Indicates statistically significant differences (<0.05) between groups as determined by one-way ANOVA (Tukey’s pair-wise comparison). Scale bar: 100 µm (*A–I*).

**Figure 2:**
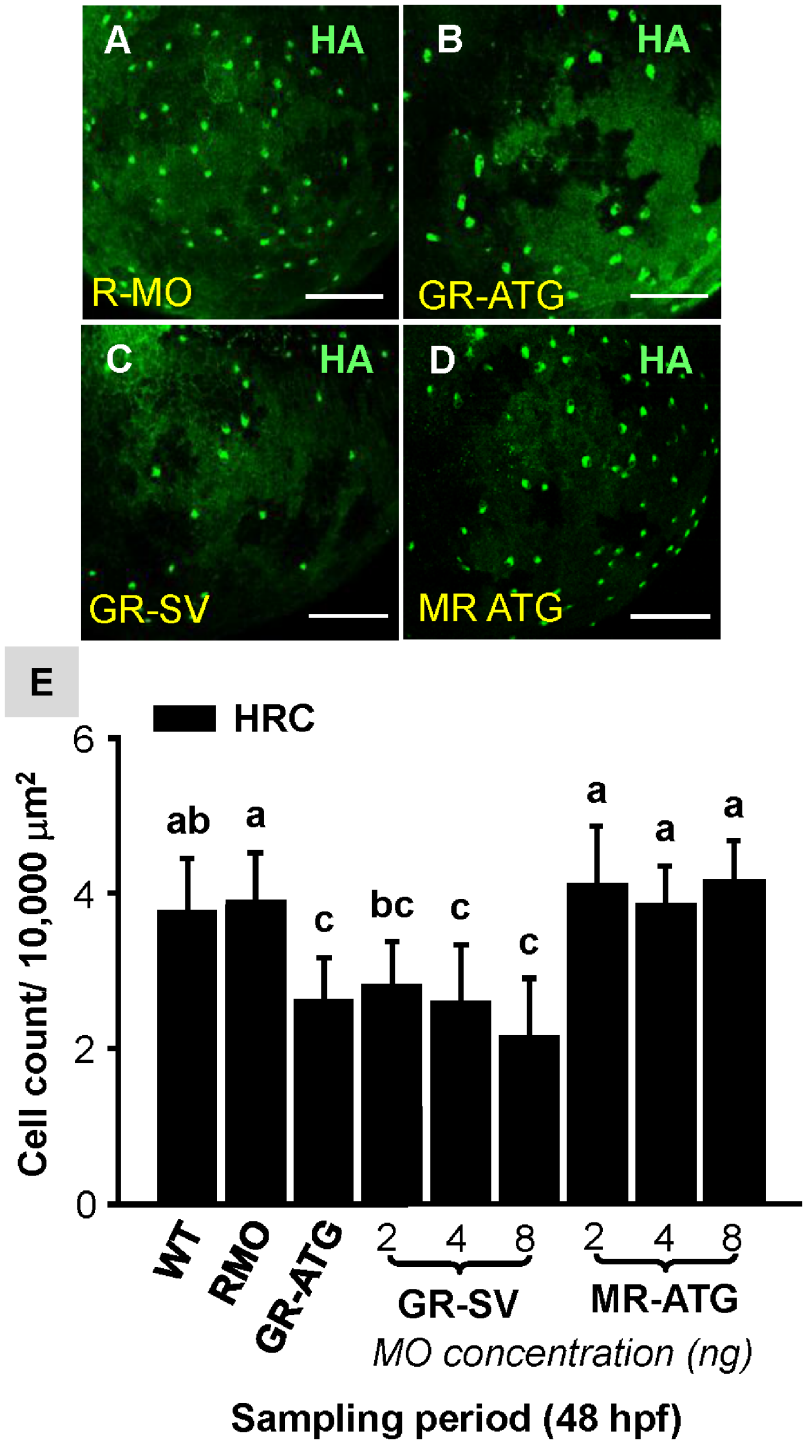
Effect of corticosteroid receptor gene knockdown on HRC number. Zebrafish embryos at the 1∼4 cell-stage were microinjected with glucocorticoid receptor ATG-MO (GR-ATG), GR-splice variant MO (GR-SV), mineralocorticoid receptor ATG-MO (MR-ATG), or Random-MO (RMO; control). Representative images of yolk-sac HRCs labeled with H^+^-ATPase (HA) in RMO (*A*), GR-ATG (*B*), GR-SV (*C*), and MR-ATG (*D*) morphants. HRC numbers are compared in (*E*). Values are presented as the mean ±s.d. (n = 10–12). ^ab^Indicates statistically significant differences (<0.05) between groups as determined by one-way ANOVA (Tukey’s pair-wise comparison). Scale bar: 100 µm (*A–F*).

To identify the specific pathway through which cortisol controls ionocyte development, we knocked down the *gr* and *mr* genes. We report that the number of epidermal NaRCs was significantly decreased in GR-ATG morphants (Fig. 1B, E) as compared to the control (Fig.1A, E). In addition, GR-SV MO, a less potent MO that blocks the transactivational activity of GR [46], significantly decreased the NaRC number in a dose-dependent manner (Fig. 1C, E). In contrast, MR-ATG knockdown did not significantly affect NaRC number at any concentration tested (Fig. 1D, E). Similarly, HRC number was also significantly decreased in GR-ATG and GR-SV morphants, and unaffected in MR-ATG morphants (Fig. 2A–E).

### The Effect of GR Over-expression on Ionocyte Number in GR Morphants

**Figure 3:**
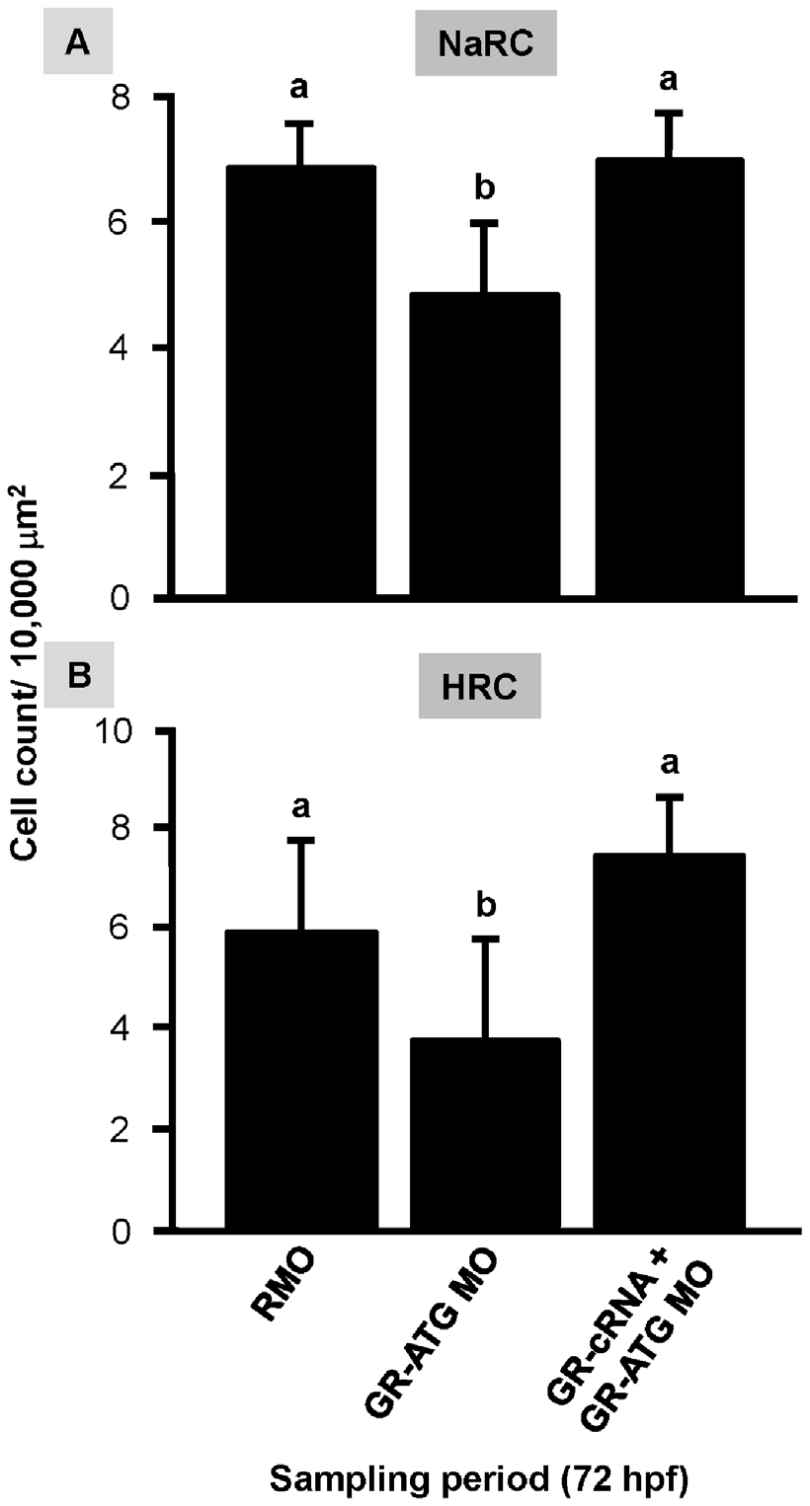
Epidermal ionocyte numbers are restored by GR rescue. Zebrafish embryos at the 1∼4 cell-stage were microinjected with glucocorticoid receptor ATG-MO (GR-ATG), GR-ATG MO plus GR-cRNA, or Random MO (RMO; control). The numbers of NaRCs (*A*) and HRCs (*B*) in yolk-sac were determined. Values are presented as the mean ± s.d. (n = 10–12). ^ab^Indicates statistically significant differences (<0.05) between groups as determined by one-way ANOVA (Tukey’s pair-wise comparison).

To confirm that the decrease in ionocytes in GR morphants was a direct result of the loss of GR, we performed a rescue experiment by over-expressing *gr* cRNA. We observed that *gr* cRNA injection rescued the effect of GR knockdown on the numbers of NaRCs and HRCs, with densities restored to a level similar to those observed in control embryos injected with the Random MO (Fig. 3A–B). These results validate the specificity and effectiveness of the GR MOs used.

### The Effect of Exogenous Cortisol Treatment on GR Morphants

**Figure 4:**
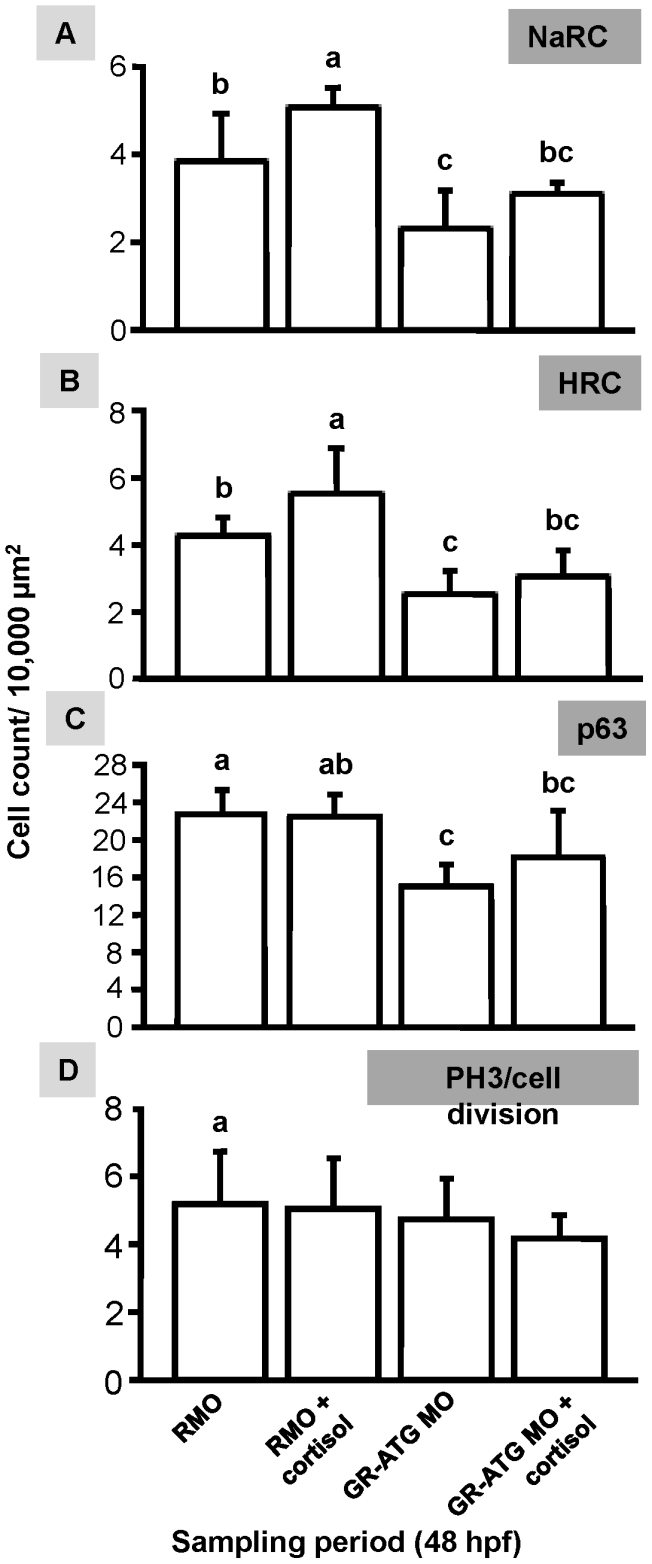
Effect of cortisol treatment on epidermal stem cells, ionocytes, and cell division in GR morphants. Morpholino-injected zebrafish embryos were reared in ambient water with or without cortisol (20 mg/L) for the entire duration of the experiment. NaRCs (*A*), HRCs (*B*), epidermal stem cells (*C*) and PH3/cell division (*D*) were counted in the yolk-sac of embryos injected with Random-MO (RMO) or GR-ATG MO in the presence or absence of cortisol (20 mg/L). Values are presented as the mean ± s.d. (n = 10–12). ^ab^Indicates statistically significant differences (<0.05) between groups as determined by one-way ANOVA (Tukey’s pair-wise comparison).

We recently reported that exogenous cortisol treatment increases the number of ionocytes [44]. To determine whether exogenous cortisol can rescue the decrease in ionocyte number induced by GR knockdown, we treated GR-ATG morphants embryos with 20 mg/L of exogenous cortisol. The number of ionocytes in cortisol-treated GR morphants was similar to that in the control group (Fig. 4A–B), which may indicate that cortisol affects ionocyte development through pathways other than Foxi3a/−b. On the other hand, epidermal stem cell number and cell division in GR morphants were unaffected by cortisol treatment (Fig. 4C–D). This result demonstrates that neither exogenous cortisol nor GR loss affect ionocyte number via the rate of epidermal stem cell division.

### The Effect of GR and MR Knockdown on the Density of Other Epidermal Cells

**Figure 5:**
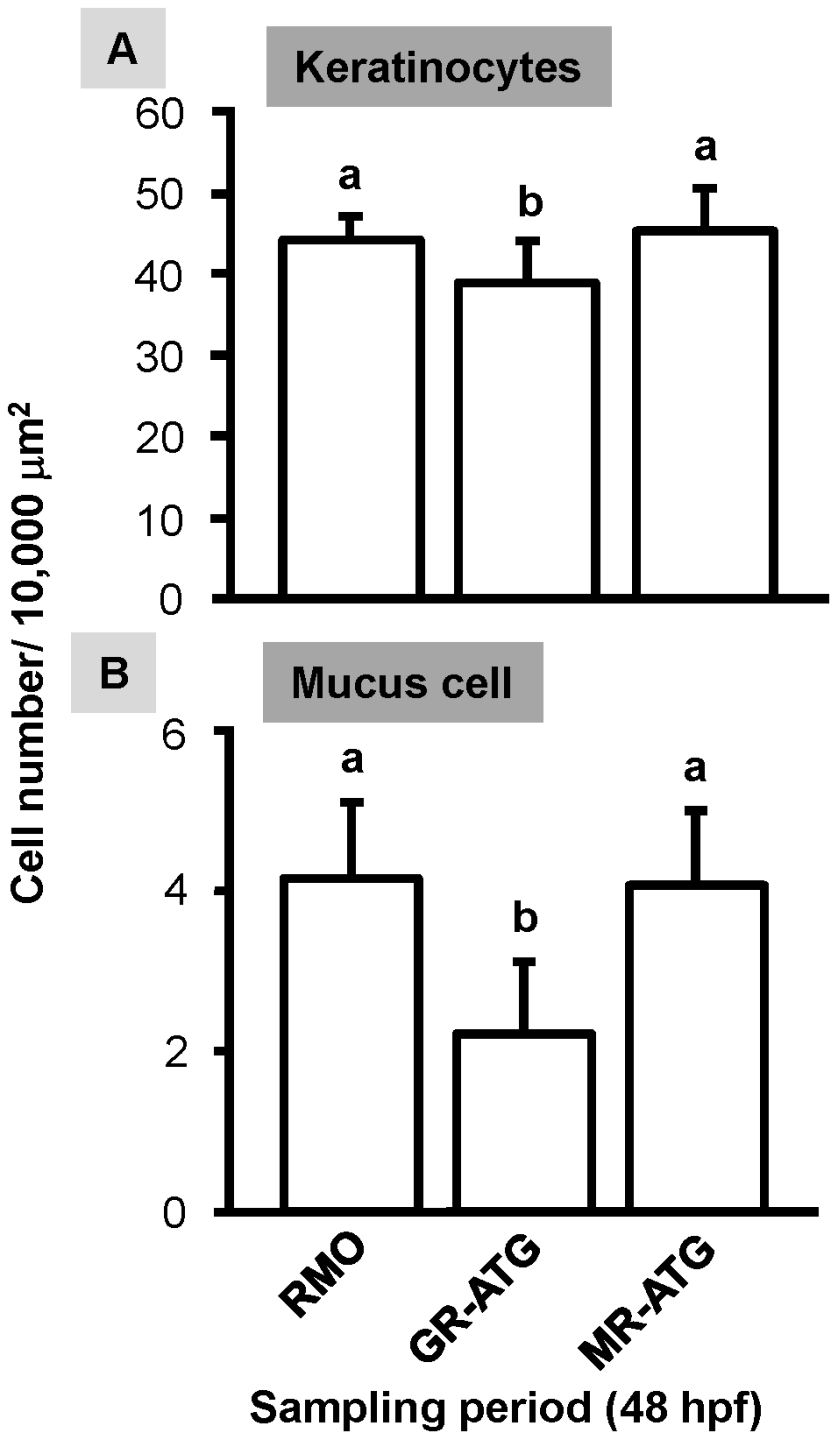
Effect of corticosteroid receptor gene knockdown on the densities of epidermal keratinocytes and mucus cells. Zebrafish embryos at the 1∼4 cell-stage were microinjected with glucocorticoid receptor ATG-MO (GR-ATG), mineralocorticoid receptor ATG-MO (MR-ATG), or Random-MO (RMO; control). Keratinocyte (*A*) and mucus cell (*B*) densities. Values are presented as the mean ± s.d. (n = 10–12). ^ab^Indicates statistically significant differences (<0.05) between groups as determined by one-way ANOVA (Tukey’s pair-wise comparison).

In addition to ionocytes, epidermal stem cells also differentiate into keratinocytes and mucus cells [40], [43]. We recently reported that exogenous cortisol treatment decreased the number of keratinocytes, but did not affect mucus cell density [44]; here, we proceeded to examine whether knockdown of GR or MR affects these cells. We report that the densities of keratinocytes and mucus cells were significantly decreased in GR morphants, but unaffected in MR morphants (Fig 5A–B). Therefore, GR may be required for the development of keratinocytes and mucus cells.

### The Effect of GR and MR Knockdown on Ca^2+^ Influx, H^+^ Secretion and Na Content in the Zebrafish Embryo

**Figure 6:**
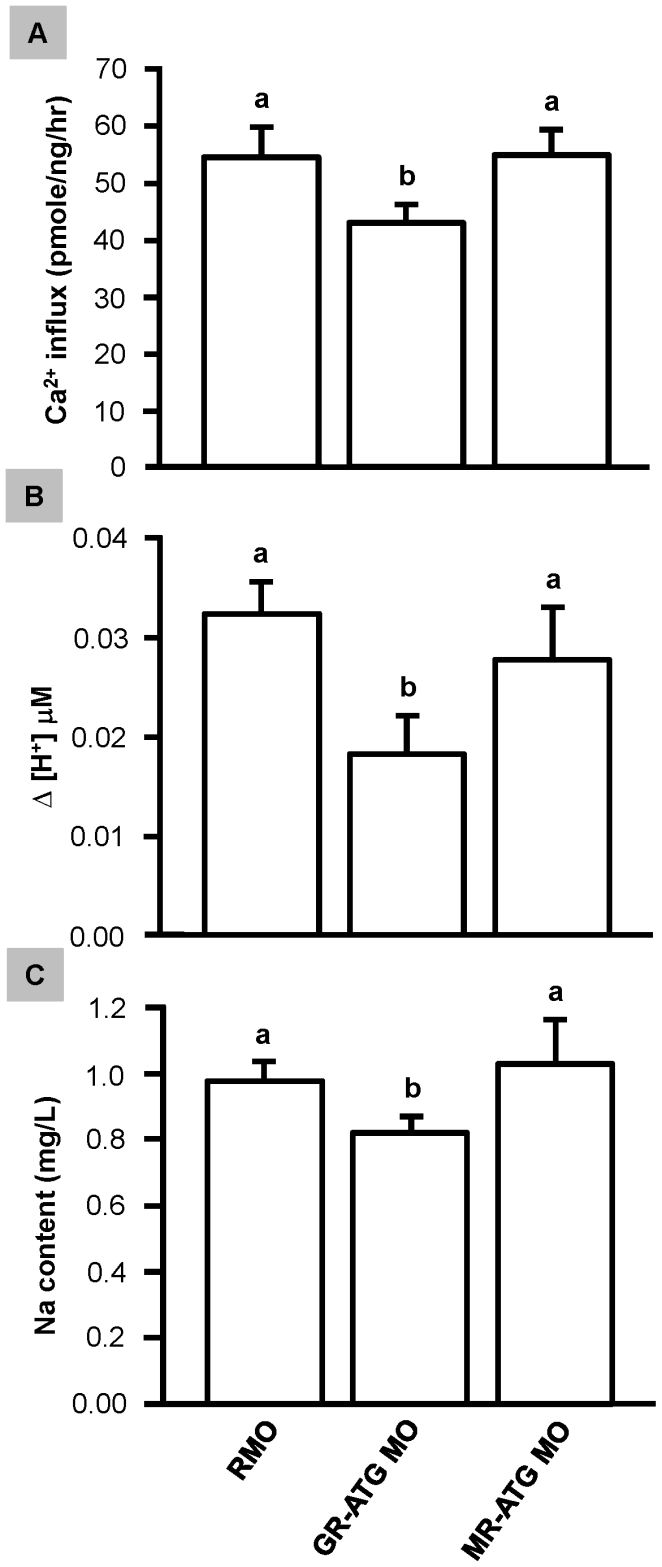
Effect of corticosteroid receptor gene knockdown on Ca^2+^ influx, acid secretion and Na content. Zebrafish embryos at the 1∼4 cell-stage were microinjected with GR-ATG MO, MR-ATG MO, or Random-MO (RMO; control). The whole embryonic Ca^+^ influx (*A*), H^+^ gradient at the embryonic skin (*B*) and Na content *(C)* were measured. All values are presented as the mean ± s.d. (n = 10). ^ab^Indicates statistically significant differences (<0.05) between control and morphants, as determined by one-way ANOVA (Tukey’s pair-wise comparison).

Previous studies have reported that GR and MR knockdown in zebrafish indicate that GR controls the functional regulation of ionocytes [26], [45]. Using a similar strategy to these earlier studies, we tested the hypothesis that the decrease in ionocytes in GR morphants would disrupt ionocyte function (NaRC-mediated Ca^2+^ influx and HRC-mediated H^+^ secretion). As predicted, Ca^2+^ influx and H^+^ secretion were significantly decreased in GR morphants, but unaffected in MR morphants (Fig. 6A–B). To further demonstrate the function of HRC in Na regulation, our results showed significant decrease in total sodium content in GR morphants but not in MR morphants (Fig. 6C). Therefore, ionocyte density is related to its functional capacity.

### Localization of GR mRNA and Protein in the Epidermal Layers of Zebrafish Embryos and Adult Gill Sections

**Figure 7:**
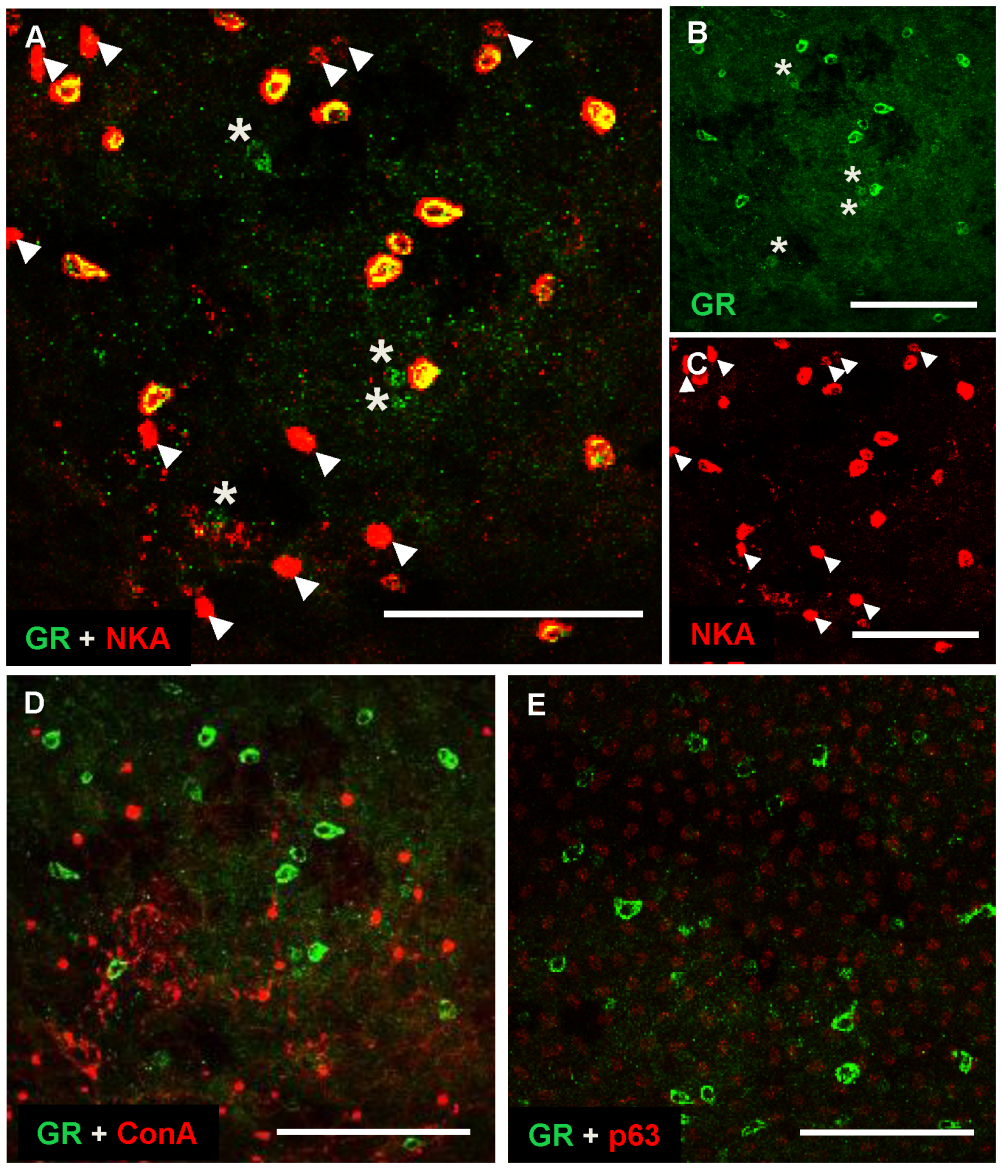
Localization patterns of GR protein in epidermal cells of zebrafish embryos. Representative images of the yolk-sac of wild-type embryos (48–96 hpf) labeled with anti-glucocorticoid receptor (GR) and anti-α sub-unit of N^+^-K^+^-ATPase (NKA) (a marker for NaRCs) (*A*), anti-GR (*B*), anti-NKA (*C*), anti-GR and ConA (a marker for HRCs) (*D*) and anti-GR with anti-p63 (a marker for epidermal stem cells) (*E*). Arrow heads indicate NaRCs without a GR signal; asterisks indicate GR-expressing cells without an NKA signal (*A–C*). Scale bar: 100 µm (*A–E*).

**Figure 8:**
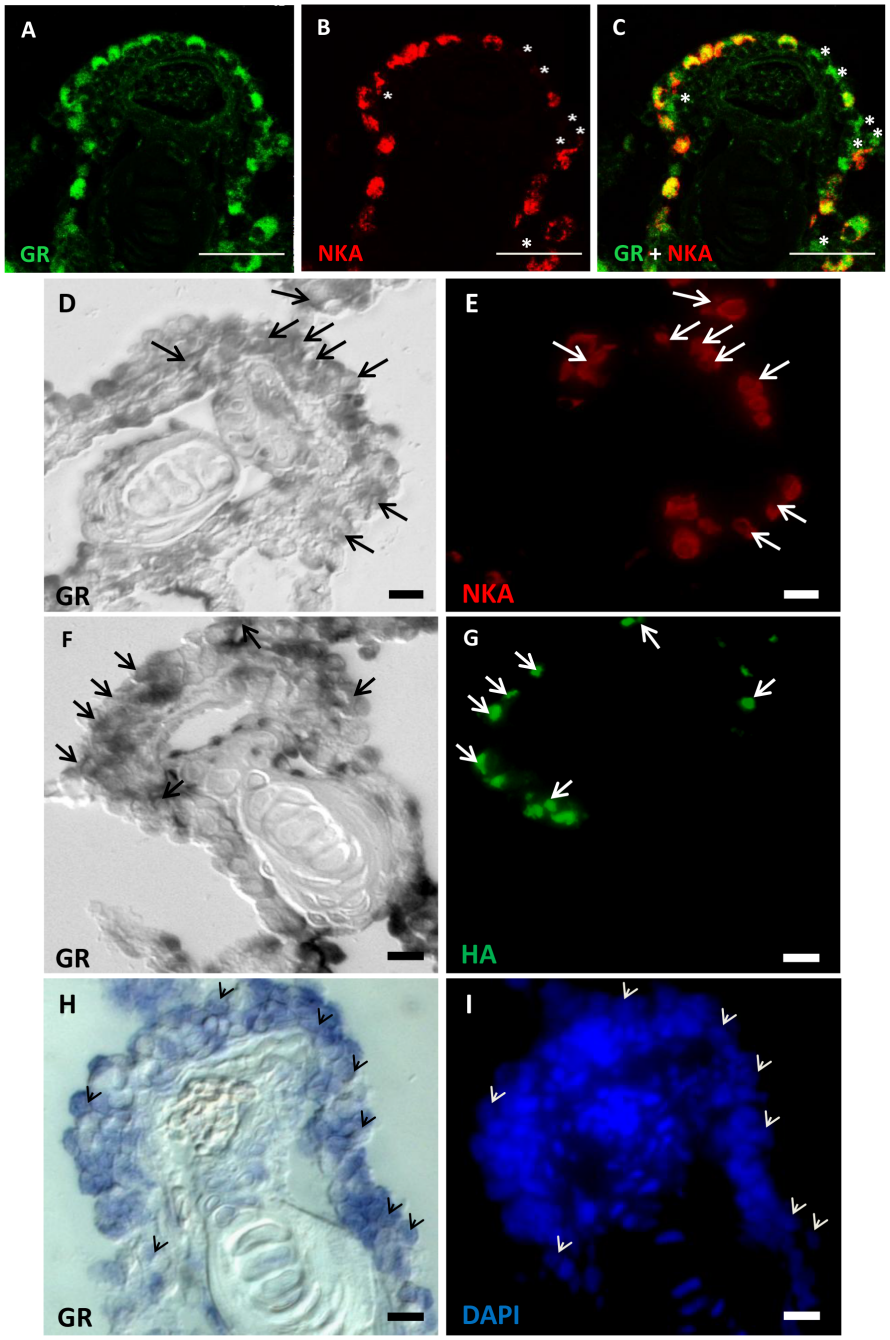
Expression patterns of GR mRNA and protein in the ionocytes of adult zebrafish gills. Representative images of gill paraffin sections labeled with anti-glucocorticoid receptor (GR) (*A*), anti-alpha sub-unit of N^+^-K^+^-ATPase (NKA) (a marker for NaRCs) (*B*), and anti-GR and anti-NKA (*C*). Asterisks indicate *gr*-expressing cells without an NKA signal (*B–C*). Gill cryosections reveal co-localization of *gr* mRNA with NKA (*D–E*) and anti-H^+^-ATPase (HA) (*F–G*). After *gr* mRNA in situ hybridization, nuclear/cellular structure can be visualized with DAPI signals (H–J). Arrows and arrow-head indicate cells with colocalized signals (*D–G*). Scale bars: 25 µm (*A–C*) and 5 µm (*D–I*).

The results above indicate that cortisol affects epidermal cell development through the GR, but not the MR. To further confirm the presence of this pathway, we examined the expression of *gr* mRNA and GR protein in epidermal cells. In 2–4 dpf zebrafish embryos, GR protein was expressed in NKA-labeled cells (NaRCs) (Fig. 7A–C); however, conA-labeled HRCs (Fig. 7D) and p63-labeled ESCs (Fig. 7E) did not exhibit an anti-GR signal. The vast majority (99%) of cells with an anti-GR signal was NaRCs; however, some NaRCs did not express GR, and some GR-expressing cells were not NaRCs. We also examined GR expression in paraffin sections of adult zebrafish gill, and observed a similar co-localization of GR in NKA-labeled NaRCs (Fig. 8A–C); again, a few GR-labeled cells did not exhibit anti-NKA signals. In addition, p63-labeled ESCs were also observed to co-express GR at a low level (data not shown). Anti-GR expression in gill sections is apparently ubiquitous, especially at a higher antibody titer (data not shown). We also observed *gr* mRNA in ionocytes doubled-labeled with immunostaining marking NaRC and HRC within cryosections. *gr* mRNA is highly expressed in a ubiquitous manner, and co-localizes with NKA (Fig 8D–E) and HA (Fig. 8F–G), indicating that both NaRC and HRC synthesize and utilize GR. Each *gr*-expressing cell was labeled with DAPI (Fig. 8H, I), and the chondrocytes (without *gr* mRNA) were also labeled with DAPI (Fig. 8H, I). As reported previously [45], absence of staining in the control images using *gr* sense-probe was observed (data not shown), confirming the validity and specificity of the assay.

## Discussion

In the present study, we provide molecular evidence that GR, but not MR, controls epidermal ionocyte development and function. We used an RNA probe specific to zebrafish *gr* to show that GR mRNA is present in most of the epithelial cells of gills, including the NaRCs and HRCs, and this is inconsistent with the protein labeling data by using a heterologous antibody designed against human GR. Nonetheless, our knockdown and functional assays validate the major involvement of GR in the development of NaRCs and HRCs, and in affecting their respective roles in calcium uptake and H^+^ secretion.

The corticosteroid signaling pathway via the hypothalamus-pituitary-interrenal (HPI) axis is active from early zebrafish development, and all of the required components are present around the time of hatching [47]–[51]. Transcripts encoding cortisol and its associated receptors are maternally deposited [52], [53], an indication of major developmental necessity. Unlike other teleosts, the zebrafish genome contains only one *gr* gene that produces two spliced variants, called *grα* and *grβ* [52], [53] as there are fewer possible receptors through which cortisol can exert an effect in zebrafish, it is a simpler task to identify the receptor of interest. These protein isoforms have only been identified in zebrafish and human, and few studies have addressed their functions. The GRβ isoform acts as a dominant-negative inhibitor of GRα, but GRβ does not have transactivational activity since it lacks the transactivation domain (AF-2) [53]. In this study, we knocked down both splice variants by using antisense GR-ATG MO, which resulted in a highly significant decrease of mature ionocytes (NaRCs and HRCs). In addition, the GR-SV MO exhibited a dose-dependent effect, indicating that impaired transactivation of GR may affect genes involved in ionocyte development, such as the genes encoding the Foxi3 transcription factors [44]. We have previously shown that exogenous cortisol stimulates ionocyte differentiation through Foxi3, and that the Foxi3a/−b promoter regions contain glucocorticoid response elements (GREs) [44], [45]. The present study further describes the signaling pathway, with cortisol apparently influencing ionocyte development by activating the Foxi3 transcription factors through the GR.

In the present study, other epidermal cells, including stem cells (marked by anti-p63) were affected by loss of GR, in contrast to our previous report that cortisol treatment did not affect ESC number. We hypothesize that treatment with exogenous cortisol within a physiologically acceptable dosage may trigger a systemic counter-regulatory effect, to maintain homeostasis. Furthermore, the treatment only affected ionocyte differentiation, which is a highly sensitive physiological process [44]. The reduction in ESCs induced by GR knockdown indicates a stronger global effect than cortisol treatment, which may have had negative effects on other epidermal cells; in addition, this finding suggests that ESCs contain GRs. This may also hold true for mucus cells, which were affected by GR knockdown, but not by exogenous cortisol treatment [44]. Furthermore, ESCs continuously undergo cell division to maintain the skin [54], but neither exogenous cortisol nor loss of GR affected cell proliferation. Therefore, we suggest that GR may be distributed ubiquitously in epidermal tissues, and it may be involved in biological processes other than regulating ionocyte development.

Previous studies reported ubiquitous expression of *gr* mRNA in zebrafish embryos [53], in most cells of adult zebrafish gills [45], and tilapia gills, kidney, and intestines [55]. Likewise, we observed ubiquitous expression of *gr* mRNA in most cell types of the gill epithelium of adult zebrafish, including ESCs (data not shown), ionocytes (NaRC and HRC), and other unidentified cells (Fig. 8). It is possible that the presence of GR in epidermal cells is common to all vertebrates, considering its high biological importance. We therefore used a commercially-available heterologous anti-GR antibody to show that GR is highly expressed in NaRCs, but not HRCs, in zebrafish embryos (tested at 2–4 dpf), a result that is the exact opposite of that of a previous study: an anti-GR signal was detected exclusively in the HRCs of 4 dpf zebrafish embryos, and the authors suggested that GR in NaRCs may be too low to be detected by a standard immunostaining protocol [26]. ]. Although we observed that GR was expressed in NaRCs in adult gill-sections, the inconsistencies in the embryonic data between studies may reflect some subtle differences in the methodologies and/or unknown artifacts. As we have followed standard immunocytochemistry techniques similar to the previous study [26], we suggest that extra precautions should be taken when considering data obtained using heterologous antibodies. Nevertheless, our data on *gr* mRNA is consistent with that of previous studies [45], [53], [55], and are thus more reliable as compared to the anti-GR ICC results.

In euryhaline fish, cortisol activity contributes to the osmo- and iono-regulation required for adaptation and survival in a fluctuating environment [21], [22], [28], [56]. It has been well documented that cortisol exerts its activity by increasing the number of ionocytes, and by stimulating the transcription, translation, and activity of ion transporters [32], [39], [44], [45], [57]–[63]. Whether these control pathways are mediated by GR, MR or both has been a long-term subject of debate, one that has been investigated primarily by pharmacological approaches; however, the evidence accumulated to date has remained inconclusive due to its conflicting nature. In freshwater-acclimated Atlantic salmon, both RU486 (a GR antagonist) and spironolactone (an MR antagonist) suppressed cortisol-mediated stimulation of gill Na^+^-K^+^-ATPase α1a mRNA *in vitro* [23] but only RU486 had an inhibitory effect *in vivo* [25]. A similar conflict was found in the case of cortisol-stimulated Atlantic salmon gill Na^+^-K^+^-ATPase α1b mRNA [23]. Both RU486 and spironolactone decreased cortisol-stimulated gill Na^+^-K^+^-ATPase α1b mRNA *in vitro* in seawater striped bass [24] and seawater Atlantic salmon [23], but only RU486 suppressed that in seawater tilapia [24]. These inconsistencies may be ascribed to differences in species or experimental design, or possibly other, unknown reasons. While spironolactone was used as MR antagonist, it also affects other biological processes that may mask the specific contribution of MR, thus limiting its use [64]. Pharmacological approaches alone are clearly insufficient at resolving the exact pathway by which cortisol exerts its action on ion regulation and ionocyte development. By exploiting the advanced molecular physiological techniques available in the zebrafish model, it may be possible to provide conclusive and convincing answers to this issue. Recent studies applying such advanced methods established zebrafish as a comprehensive working model of ionocyte development, function, and functional regulation [28], [42], [43]. In the proposed model, different types of identified ionocyte express ion transporters according to their known functions; NaRCs express ECaC for Ca^2+^ uptake, whereas HRCs express HA and Na^+^/H^+^exchanger (NHE3b) for H^+^ secretion and Na^+^ transport, respectively [65]–[70]. A recent loss-of-function approach using specific GR and MR morpholinos demonstrated that only GR knockdown caused *ecac* mRNA to decrease, which eventually impaired Ca^+^ uptake in zebrafish embryos [45]. We used a similar knockdown technique to confirm the decrease in NaRC density and Ca^2+^ uptake in zebrafish GR morphants. On the other hand, it was reported that Na^+^ uptake (via HRC) in zebrafish embryo was compromised upon knockdown of GR, but not in the treated embryos of a selective MR agonist, aldosterone [26]. In agreement, we found that HRC density, and consequently, epidermal H^+^ secretion and sodium content were decreased in GR, but not MR, morphants. Hence, GR knockdown using gene specific morpholino oligonucleotides has clearly demonstrated that the GR plays a major role in ionocyte development and function. In medaka, GR, but not MR, was also shown to be involved in the regulation of ionocyte differentiation in the embryonic skin (V. Trayer, P. P. Hwang, P. Prunet, V. Thermes, unpublished data). Whether this holds true in other species awaits confirmation.

Salinity acclimation experiments in rainbow trout revealed that levels of *mr* mRNA exhibit tissue-, salinity- and time-dependent changes, without accompanying changes in the levels of DOC or DOC-mediated effects on gill ionic-related transporters; this suggests a major MC role for cortisol [20]. In mammals, cortisol has been known as the major ligand of the MR in the heart and central nervous system [5]. But the epithelial cells of the mammalian kidney and intestine contain high levels of 11-β-hydroxysteroid dehydrogenase 2 (11βHSD2), which converts GC into an inactive state; this facilitates preferential binding of aldosterone to the MR in these cells [71]–[74]. As previously suggested, this pathway may also exist in fish [20], and DOC-MR selectivity may also be present in other zebrafish tissues. Our data indicate that ionocyte density is significantly decreased upon GR knockdown but unaffected by MR knockdown; moreover, higher doses of MR-ATG MO actually resulted in a significant increase in NaRCs (data not shown). In response to this finding, we suggest that the absence of MR may enable increased GR-selectivity by reducing ligand-binding competition between the CR receptors. Furthermore, aldosterone treatment did not affect Na^+^ uptake in zebrafish [26], and MR knockdown did not affect Ca^2+^ uptake [45]. Together, these studies provide unanimous evidence that MR is not involved in these processes, and we hypothesize that levels of DOC, MR, and 11βHSD2 may be too low to participate in zebrafish embryo skin development and ionoregulation.

## Conclusions

Extensive investigation has demonstrated that the role of MC in mammals in controlling osmo- and iono-regulation mechanisms can be performed by cortisol in fish. Here, we have provided convincing molecular evidence that cortisol regulates the differentiation of skin epidermal cells (specifically ionocytes) through the GR only, and thus acts in a similar manner to mammalian GC. This further reinforces the previously-held view that in fish, which lack aldosterone, cortisol performs the functions ascribed to GC and MC in mammals. Hence, we have provided a new platform to facilitate studies into the dual role of GC through GR in skin/gill epidermal development and ionoregulation, which promises to resolve the ambiguous findings of the past.

## Materials and Methods

### Experimental Animals

Adult zebrafish (*Danio rerio*) were obtained from stocks held at the Institute of Cellular and Organismic Biology, Academia Sinica, Taipei, Taiwan. Fish were reared in a circulating system containing local fresh water at 28±0.5°C under a 14∶10 h light: dark photoperiod. Daily feeding consisted of artificial feed pellets (Fu-So, Taipei, Taiwan). The Academia Sinica Institutional Animal Care and Utilization Committee approved all the experimental protocols used in this study.

### Knockdown with Morpholino Antisense Oligonucleotides

We utilized similar morpholino oligonucleotides (MOs) to those reported previously [45], and these were designed and generated by GENE Tools (Philomath, OR, USA). Antisense MOs were designed to target the start codon (ATG) of *gr* (GR-ATG MO, 5′-CATTCTCCAGTCCTCCTTGATCCAT-3′) and *mr* (MR-ATG MO, 5′ ACGACATCCGATTTTGACAGTTACC-3′). A random MO (R-MO, 5′-CCTCTTACCTCAGTTACAATTTATA-3′) was used for the control group. Another MO, GR-SV, was designed against the *gr* splicing variant (5′ CTGCTTCATGTATTTTAGGGTTCCG-3′), similar to previous reports [46]. MOs were resuspended in Danieau buffer. The efficacy of each MO was examined at different doses (1, 2, 4, and 8 ng per embryo). GR-ATG-MO caused severe abnormalities at higher doses, and 2 ng is the maximum dose at which normal development can proceed, while 4 ng was utilized for MR-ATG MO for all the succeeding experiments similar to previous study [45]. The specificity of GR-ATG MO and MR-ATG MO were examined using partial sequences spanning the MO target site inserted into pCS2+ vector upstream of a green fluorescent protein (GFP) construct (data not shown), also demonstrated previously [45]. Further confirmation by western blot previously [45], whole-mount proteins collected from control MO, GR-ATG MO, and MR-ATG-MO morphants were labeled with anti-GRα and anti-MR polyclonal antibodies (raised in rabbit against the relevant human protein). In addition, we have analysed the western blot protein relative abundance using image processing program (ImageJ 1.45 s), showing no significant difference between GR-ATG-MO and MR-ATG-MO knockdown (relative percentage of blocked translation by MO normalized using the control MO: GR-ATG-MO 75±15% [N = 4]; MR MO 62±11% [N = 4]). The partial knockdown of GR or MR was to ensure normal developmental/morphological processes. The specificity of GR-SV MO was previously established [46]. Rescue experiments were performed using a pCS2 vector containing the full *gr* gene sequence except for the stop codon. Primer sequences are listed in Table 1. Capped mRNAs (cRNAs) encoding *gr* were synthesized from the vector using the SP6 mMessage mMachine kit (Ambion, Austin, TX, USA), and the sequences were checked before use. We injected GR cRNAs at test concentrations between 10–300 pg per embryo, revealing that 50 pg could be safely injected with or without the MO at the 1∼ 2 cell stages. Embryos were incubated at 28°C for further observation.

**Table 1:** Primers used for qPCR analysis and PCS2: GR cloning.

Gene	Accession number	Sequence 5′-3′
*gr*	**NM_001020711**	*Forward* ACAGCTTCTTCCAGCCTCAG *reverse* CCGGTGTTCTCCTGTTTGAT
*mr*	**NM_001100403**	*forward* ACAGAGGCAACAATGATTAGAG *reverse* GTTCTCCCACAAAGAGGGT
*β-actin*	**NM_131031**	*forward* ATTGCTGACAGGATGCAGAAG *reverse* GATGGTCCAGACTCATCGTACTC
PCS2: GR		*forward* CATCGATTCGAATTCAATGCAAAATGGATCAAGGAGG *reverse* TCAATGTATCTTATCATGTGATGAAAGAGCAGCGGTTTAA

### Cortisol Treatment of Zebrafish Embryos

We treated MO morphant embryos with exogenous cortisol, as described below. A dose of 20 mg/L hydrocortisone was previously reported to be efficient and stable at affecting epidermal ionocyte number and function in zebrafish, without disturbing the growth rate of the developing embryos [44], [45]. We accordingly chose a similar dosage of hydrocortisone-21-hemisuccinate (Sigma) for use in our experiments. Newly-fertilized zebrafish eggs were grown in 20 mg/L hydrocortisone-21-hemisuccinate (tap water) with or without MO injection, and then incubated in a controlled system at 28°C together with the control group (tap water only). At the end of the experiment, samples were fixed for further analysis.

### Immunocytochemistry (ICC) of Whole-mount Zebrafish Embryos and Adult Gill Sections

Following gene knock-down and/or cortisol treatment, embryos were fixed with 4% paraformaldehyde in phosphate buffered saline (PBS) solution for 2 h. After fixation, samples were washed with PBS twice, and stored in 100% methanol at −20°C for future use. Prior to ICC, samples were washed twice in PBS containing Tween 20 (PBST), and incubated in 100% ethanol for 15 mins at −20°C. Samples were then washed several times in PBS and blocked using a PBST solution containing 2% sheep serum and 2 mg/ml BSA for 4 h at room temperature. Samples were subsequently incubated at 4°C overnight with one of the following primary antibodies; anti-p63 monoclonal as a marker for ESCs; anti-avian Na^+^-K^+^-ATPase (NKA) α-subunit monoclonal for NaRCs (α 5, 1∶500 dilution; Developmental Studies Hybridoma Bank, University of Iowa); anti-α subunit of killifish H^+^-ATPase (HA) polyclonal for HRCs (1∶200 dilution) [75]; anti-cytokeratin monoclonal for keratinocytes; and anti-phosphorylated Ser-10 of histone 3 (PH3) polyclonal to monitor cell division. A previously reported purified polyclonal antibody raised against the C-terminus of human GR α l (Santa Cruz Biotechnology, Inc.) was used at a titer of 1∶100–200 [26]. All antibodies were diluted in blocking PBST solution. After incubation with the primary antibody, samples were washed several times with PBST, and then incubated with secondary antibody (goat anti-rabbit/mouse IgG conjugated with Alexa Fluor 568 or 488 (1∶300 dilution; Molecular Probes)) for 2 h in blocking solution at room temperature. Samples were washed several times with PBST, and then examined under a confocal laser scanning microscope (TCS-SP5, Leica Lasertechnik, Heidelberg, Germany).

### Alcian Blue Staining of Mucus Cells

Mucus cells were stained after gene knockdown, as previously described [44]. Samples were fixed in 4% paraformaldehyde for 6–7 d at 4°C, and then washed several times with PBS for 5 min each. All subsequent steps were performed at 37°C. Samples were rinsed with acid ethanol (70% ethanol plus 0.37% HCl) for 5 min, and then stained with 0.1% alcian blue 8GX (Sigma) solution in acid alcohol for 3 h. Samples were sequentially washed in decreasing concentrations of acid ethanol, down to PBS. Samples were incubated with 1% trypsin in PBS for 1 h, and then washed several times in PBS. Samples were stored in 75% glycerol prior to image acquisition.

### Measurement of Ca^2+^-uptake in Zebrafish Embryos

Dechorionated zebrafish embryos were briefly rinsed in deionized water before being transferred to 2 ml of medium containing ^45^Ca^2+^ (Amersham, Piscataway, NJ, USA) with a final working specific activity of 1∼2 mCi/mmol, and incubated for 4 h at 28±0.5°C. Samples were subsequently washed several times using freshwater without the isotope. Embryos were anesthetized with 0.2% buffered MS-222, and six embryos were then pooled into one vial before being digested using a tissue solubilizer (Solvable; Packard, Meriden, CT, USA) at 60°C for 8 h. Digested solutions were added to counting solution (Ultima Gold; Packard), and radioactivity was analysed using a liquid scintillation beta counter (LS6500; Beckman, Fullerton, CA, USA). Ca^2+^ influx was calculated using the formula previously described [76].

### Measurement of Surface H^+^-secretion Using Scanning Ionselective Electrode Technique (SIET)

SIET was used to measure the extracellular H^+^-efflux concentration at the surface of live zebrafish larvae, as an indication of the major function of HRCs. H^+^-selective microelectrodes were constructed as previously described [65], [77]: briefly, glass capillary tubes (no. TW 150-4, World Precision Instruments, Sarasota, FL) were pulled using a Sutter P-97 Flaming Brown pipette puller (Sutter Instruments, San Rafael, CA), and were used as micropipettes with tip diameters of 3–4 µm. Glass micropipettes baked at 120°C overnight were vapor-silanized with dimethyl chlorosilane (Fluka, Buchs, Switzerland) for 30 min. Prior to usage, micropipettes were backfilled with a 1 cm column of 100 mM KH_2_PO_4_, thereby creating a H^+^ microelectrode. Micropipettes were subsequently front-loaded with a 20- to 30-µm column of liquid ion exchanger cocktail (H^+^ ionophore I *cocktail B*; Fluka). The Nernstian property of each microelectrode was analysed using a series of standard solutions (pH 6, 7, and 8). The plot of the probe voltage output against the log H^+^ concentrations yielded a linear regression with a Nernstian slope of 57.5±2.5 (*n = *10) for H^+^.

SIET was executed in a small plastic recording chamber filled with 1 ml recording medium (artificial freshwater with 300 µM MOPS buffer: Sigma, St. Louis, MO), and 0.1 mg/L ethyl 3-aminobenzoate (Tricaine, Sigma; pH 7.0) at room temperature (26–28°C). H^+^ activity was recorded at the surface of the larva, with the microelectrode placed at a target position 10–20 µm away from the skin. After recording at each target point, the microelectrode was moved away (∼10 mm) to record the background. The Δ[H^+^] value represents the measured H^+^ gradients between the point of interest (skin surface) and the background.

### Whole-body Sodium Content Measurement

Total of fifteen zebrafish larvae were pooled as one sample (5 replicate) and briefly rinsed in deionized water. The samples were digested by HNO3 at 13.1 N at 60°C overnight. Double-deionized water was used to dilute the digested solutions, and the total sodium content was measured using atomic absorption spectrophotometry (Z-8000; Hitachi, Tokyo, Japan). Sodium standard solution from Merck (Darmstadt, Germany) was used to make the standard curves measurement.

### Double-labeling of GR mRNA and Protein in Adult Zebrafish Gills

Gill paraffin sections and cryosections were acquired as previously described, with a few modifications [55], [78]. GR primer sets were obtained as previously reported, and sense probes were tested to establish the specificity of anti-sense probes [45]. *Gr* transcripts were subjected to RT-PCR and the resulting amplicon was ligated into pGEM-T easy vector, before being used to transform *E*. *coli* (Promega, Madison, WI, USA). After confirmation of the target plasmid DNA sequence, the *gr* transcript was amplified using T7 and SP6 primers by RT-PCR, and the amplicons were used as templates for *in vitro* transcription with T7 or SP6 RNA polymerase and digoxigenin (DIG)-UTP (Roche) to synthesize GR riboprobes. Double-labeling of GR mRNA and protein by *in situ* hybridization and antibody staining was performed as described previously [45], [78]. The following antibodies were used; anti-NKA, anti-HA, anti-p63 and anti-GR. The 4′, 6-diamidino-2-phenylindole (DAPI) was used to stain nuclear DNA and RNA in gill cryosection for a better visualization of each cell. All images were acquired using a bright-field microscope with a digital camera (Leica DFC420 C, Leica Microsystems, CH-9435 Heerbrugg, Germany).

### Statistical Analysis

Values are presented as the mean ± standard deviation (S.D.). Differences among groups were identified by one-way analysis of variance (ANOVA) (Tukey’s method). A significant difference was accepted at (*) p<0.05.
